# Initial outcome of contrast-enhanced spectral mammography in lesion characterisation in the mammographically dense breast

**DOI:** 10.1186/bcr3780

**Published:** 2015-11-05

**Authors:** Zeiad Hussain, Peter Duggan, Julie Scudder, Sarah McWilliams

**Affiliations:** 1 Guys and St Thomas NHS Trust, London, UK

## Introduction

The accuracy of mammography is limited in the dense breast. Contrast-enhanced spectral mammography (CESM) is a relatively new alternative with promising results. The aim of this study is to assess the sensitivity, specificity, positive predictive value (PPV) and negative predicative value (NPV) of CESM in the detection and characterization of breast lesions.

## Methods

Retrospective review of the prospectively maintained database of patients who underwent CESM over a period of 6 months. The sensitivity, specificity, PPV and NPV of CESM were assessed against the histopathology result. In a subgroup of eight patients, the CESM outcome was also compared to MRI scan results. In addition, patients' demographics and correlation with mammography and ultrasound outcomes were obtained.

## Results

Twenty-four patients (23 female) underwent CESM over a 6-month period. CESM was found to have a sensitivity of 76 %, specificity of 66 %, PPV of 93 % and NPV of 66 %. The median maximal lesion dimension (MMLD) on CESM was 29 mm. In a subgroup of eight patients MRI was also performed where the MMLD on MRI was 22 mm and on CEM was 20 mm. MRI accurately diagnosed all malignant lesions (8/8) while CEM demonstrated a false negative results in 2/8 patients. In five of the 11 patients who initially had mammography and four of the 19 patients who initially had ultrasound scan demonstrating indeterminate lesions, CEM correctly characterised the lesions.

## Conclusion

CESM is a promising and affordable technique in the assessment of suspicious lesions in the mammographically dense breast. CEM has a good sensitivity, specificity, PPV and NPV.

